# Effects of Flaxseed Oil and Vitamin E Supplementation on Digestibility and Milk Fatty Composition and Antioxidant Capacity in Water Buffaloes

**DOI:** 10.3390/ani10081294

**Published:** 2020-07-29

**Authors:** Bruna C. Agustinho, Lucia M. Zeoula, Nadine W. Santos, Erica Machado, Emerson H. Yoshimura, Jessyca C. R. Ribas, Janaina M. Bragatto, Mariana R. Stemposki, Vanessa J. dos Santos, Antonio P. Faciola

**Affiliations:** 1 Department of Animal Science, State University of Maringa, Maringa PR 87020-900, Brazil; lmzeoula@uem.br (L.M.Z.); santos.woruby.n@gmail.com (N.W.S.); ericazoo@hotmail.com (E.M.); ehy.vet@gmail.com (E.H.Y.); ribascarol@ymail.com (J.C.R.R.); janainabragatto@gmail.com (J.M.B.); mari.stemposki@gmail.com (M.R.S.); 2 Department of Chemistry, State University of Maringa, Maringa PR 87020-900, Brazil; vanessajs_11@hotmail.com; 3 Department of Animal Sciences, University of Florida, Gainesville, FL 32611, USA; afaciola@ufl.edu

**Keywords:** dairy, digestibility, fat, oxidation, tocopherol

## Abstract

**Simple Summary:**

Flaxseed oil is rich in n-3 fatty acids, while vitamin E is a potent antioxidant. Both have been tested in dairy cows’ diets to increase n-3 concentration and antioxidant capacity in the milk. However, there is no published research testing flaxseed oil and vitamin E supplementation simultaneously in lactating dairy buffaloes, which can have a different response compared to dairy cows. Increasing milk unsaturated fatty acids while not increasing lipid oxidation is a challenge; however, in this experiment we demonstrated that it is possible to achieve these in buffalo milk by supplementing the diet with flaxseed oil and vitamin E. Flaxseed oil supplementation increased the n-3 fatty acid concentration and oxidation products in the milk, while vitamin E supplementation increased milk’s antioxidant capacity.

**Abstract:**

This study aimed to evaluate the effects of the supplementation of flaxseed oil and/or vitamin E on dry matter (DM) and nutrient digestibility, milk composition, fatty acid composition, and antioxidant capacity in buffalo milk. Four crossbred female dairy water buffaloes (97 ± 22 days in milk; 6.57 ± 2.2 kg of milk/day, mean ± SD) were distributed in a 4 × 4 Latin square design, with a 2 × 2 factorial arrangement (with or without flaxseed oil at 25 g/kg dry matter; with or without vitamin E at 375 IU/kg dry matter). The experimental period was divided into four periods of 21 days each (16 days for adaptation; five days for data collection). There were four treatments: control diet (no flaxseed oil and no added vitamin E); flaxseed oil diet (flaxseed oil at 25 g/kg DM); vitamin E diet (vitamin E at 375 IU/kg DM), and a combination of both flaxseed oil and vitamin E. The animals were fed total mixed ratios. For all response variables, there was no interaction between flaxseed oil and vitamin E. Flaxseed oil supplementation reduced neutral detergent fiber (NDF) and acid detergent fiber (ADF) apparent total tract digestibility, increased the n-3 fatty acid concentration in milk approximately three-fold while reducing the n-6/n-3 ratio from 9.3:1 to 2.4:1. Vitamin E supplementation increased NDF apparent total tract digestibility and milk total antioxidant capacity. Although there was no interaction between the treatments; flaxseed oil supplementation in lactating buffaloes increased polyunsaturated fatty acid, while vitamin E supplementation increased antioxidant capacity and decreased oxidation products.

## 1. Introduction

According to Brazilian Institute of Geography and Statistics [1], Brazil has approximately 1.4 million water buffaloes, distributed across 14,853 farms specializing in milk and meat production [2]. Therefore, it plays an important role in Brazil’s agriculture. Moreover, in Brazil, buffalo milk is mostly used for cheese making, in which buffalo mozzarella cheese represents 70% of these dairy products [3].

The supplementation of lactating dairy buffaloes with flaxseed, which is rich in alpha-linolenic acid (18:3 n-3), modifies the fatty acid composition of milk fat by increasing the concentrations of polyunsaturated fatty acid (PUFA) and total conjugated linoleic acid (CLA) and reducing the n-6/n-3 ratio [4]. This reduced ratio may mitigate inflammatory reactions in the human body because n-6 and n-3 compete for the same enzyme, and the end product of n-6, arachidonic acid, promotes the inflammatory response in the body, while n-3 is a precursor of anti-inflammatory cytokines [5]. Both are important—but in balanced proportions. CLA isomer *cis*-9, *trans*-11 18:2 has been shown to have several health benefits for humans, such as anticarcinogenic effects [6]. However, PUFAs have carbon chains that are prone to lipoperoxidation, since carbon double bonds easily lose electrons through the action of free radicals and light [7].

One way to improve milk’s antioxidant capacity, which is the ability of redox molecules to eliminate free radicals [8], is to use dietary antioxidants in lactating animals [9]. Exogenous vitamin E supplementation has been shown to have great effects on milk due to increases in antioxidant capacity [10], which may reduce milk oxidation.

A major drawback of feeding PUFA to lactating cows is that PUFA may affect ruminal microorganisms and ruminal fermentation, increasing the propensity for milk fat depression and reducing animal performance [11,12]. Due to these issues, the dietary inclusion of PUFA should be limited. It is still unclear whether PUFA would have the same effects on milk fat in buffaloes.

Studies with oil and vitamin E supplementation in dairy cows have shown changes in milk fatty acid composition, including increased unsaturated fatty acid concentrations and reduced milk oxidation. On the other hand, there were increases in antioxidant capacity in diets supplemented with vitamin E, as observed by Santos et al. [10] and Fauteux et al. [13].

Changes in milk composition have been observed when diets supplemented with ingredients rich in n-3 were fed to water buffaloes [4]; however, there are no reports on the effects of combining n-3 and vitamin E in the diet on digestibility and milk composition in dairy water buffaloes.

Therefore, the objectives of this study were to evaluate the effects of dietary supplementation of flaxseed oil, vitamin E, and their combination on dry matter intake and nutrient apparent total tract digestibility, as well as changes in the antioxidant capacity, chemical, and fatty acid composition of milk in lactating water buffaloes. We hypothesized that flaxseed oil supplementation would increase n-3 concentration while vitamin E would increase antioxidant capacity and consequently decrease milk oxidation, and the association between the two treatments would increase n-3 concentration in the milk in conjunction with lower oxidation.

## 2. Materials and Methods

### 2.1. Cows, Animals, and Experimental Diets

The experiment was approved by the Committee for Use of Animals in Experimentation of the State University of Maringá, PR, and the study fully complied with the ethical principles of animal experimentation prepared by the Brazilian College of Animal Experimentation. The experiments were conducted at the Iguatemi Experimental Farm, PR, Brazil (4342160216/2015).

Four lactating crossbred water buffaloes (Murrah × Jaffarabadi; 97 ± 22 days in milk; 6.57 ± 2.2 kg of milk/day, mean ± SD), with a mean body weight of 655 ± 37 kg, were used in a 4 × 4 Latin square design with a 2 × 2 factorial arrangement (with or without flaxseed oil; with or without vitamin E (α-tocopherol acetate)).

Experimental diets were formulated to meet the nutritional requirements of water buffaloes weighing 650 kg, with a milk yield of 8 kg/day of milk, with 65 g/kg of fat, according to Paul and Lal [14]. These diets were as follows: a control diet (no flaxseed oil and no added vitamin E); a flaxseed oil diet (flaxseed oil at 25 g/kg DM); a vitamin E diet (vitamin E at 375 IU/kg DM), and a combination of both flaxseed oil and vitamin E (Table 1). The forage to concentrate ratio of the experimental diets was 700:300, on a dry matter basis.

The experimental period consisted of 84 days, and it was divided into four periods of 21 days each, with 16 days for adaptation to the treatments and five days for data collection. The animals were fed total mixed ratios, and diets were offered daily at 08:30 and 16:00 h ad libitum, allowing 50 to 100 g of refusal/kg, as fed. Buffaloes were milked once daily at 08:00 h, with the calf present.

The daily amount of flaxseed oil and vitamin E was provided at the feeding times, both divided into two doses daily, proportional to the morning and afternoon offering. Flaxseed oil was weighed and mixed in the concentrate prior to feeding. To ensure the ingestion of vitamin E (α-tocopherol acetate), this was previously weighed and added at an amount of approximately 50 g of total mixed ration. Only after the complete ingestion of this vitamin was the remaining diet offered.

### 2.2. Sampling and Measurements

The amount of feed provided and the refusals of each animal were weighed and recorded daily. Feed, refusals, and feces were sampled daily from day 17 to day 21 of each experimental period. Fecal samples were collected directly from the rectum at 08:30 and 16:30 h. All samples were stored in plastic bags and frozen at −20 °C. These samples were later thawed, dried in a forced air ventilation oven at 55 °C for 72 h, and ground in a Wiley mill (Marconi MA340, Piracicaba, Brazil) to 2 mm for the determination of indigestible neutral detergent fiber (iNDF) and to 1 mm for other chemical analyses. Fecal and refusal samples were pooled for each cow to obtain a composite sample per treatment and period. The estimation of fecal production was performed by determining the concentration of iNDF in feces, feed, and refusals to determine the apparent DM and nutrients total tract digestibility.

The animals were milked once daily and milk production was recorded. Between day 17 and day 21 of each period, the entire amount of milk from each animal was individually collected and homogenized, then milk samples were divided into two aliquots. The first aliquot, around 50 mL of the milk, was kept at room temperature and stored with 2-bromo-2-nitropropane-1,3-diol (Bronopol) for the determination of fat, protein, lactose, defatted dry extract, and milk density. The second aliquot, around 100 mL of milk, without the addition of preservative, was frozen at −20 °C for the subsequent determination of the concentration of fatty acids and antioxidant parameters.

The determination of in vitro dry matter digestibility (IVDMD) was performed according to the first stage (48 h fermentation with ruminal microorganisms) of the methodology proposed by Tilley and Terry [16] to evaluate the effects of oil and vitamin E on ruminal digestibility. Ruminal content was taken from three ruminally cannulated crossbred buffaloes (Murrah × Jaffarabadi) through ruminal cannula. Buffaloes were fed a diet formulated for the maintenance requirement (crude protein = 8%; Total Digestible Nutrients = 65%), according to Paul and Lal [14]. The diet consisted of corn silage, ground corn, soybean meal, wheat meal, and a mineral and vitamin supplement. The liquid and solid phase of the ruminal contents were collected manually prior to the morning feeding, from the ventral, central, and dorsal areas of the rumen. Ruminal contents were mixed in a blender, filtered into four layers of cheesecloth, flushed with CO_2_, and maintained at 39 °C until incubation.

Diet sample (0.5 g), artificial saliva (40 mL), and ruminal fluid (10 mL) were added into each tube. The tubes were saturated with CO_2_ to maintain an anaerobic environment, and incubations were performed in a water bath for 48 h at 39 °C with constant stirring. Duplicate samples were incubated in three in vitro runs. In each incubation, two additional tubes containing only artificial saliva and ruminal fluid, and another two tubes with a forge with a known IVDMD, were included as blank and standard, respectively. After incubation, residues were filtered in analytical filter paper N° 40 and dried to calculate the DM disappearance.

Samples (2 mm) were weighed in ANKOM^®^ F57 filter bags (Ankom Technology, Macedon, NY, USA) and incubated for 288 h in ruminally cannulated buffaloes fed a standard diet of 700 g/kg of *Cynodon nlemfuensis* pasture and 300 g/kg of concentrate formulated for buffaloes in maintenance, according to Vanzant et al. [17], to determine the iNDF concentration.

### 2.3. Chemical Analyses

The DM content of samples was determined in an oven, according to method 924.01 of the Association of Official Analytical Chemists (AOAC) [18]. Ash was determined by combustion at 600 °C for 6 h, according to method 924.05 of AOAC International [18]. The determination of total nitrogen was performed according to the Kjeldahl method, 990.03, of AOAC International [19]. Neutral detergent fiber (NDF) was determined according to Mertens et al. [20], using thermostable α-amylase without sodium sulfite. Acid detergent fiber (ADF) was determined according to method 973.18 of AOAC International [18]. Ether extract (EE) was determined according to method 7.060 of AOAC International [18].

After removal from the rumen, filter bags were washed and subjected to NDF analysis, according to Mertens et al. [20].

Fat, protein, lactose, and defatted dry extract concentrations in milk and milk density were determined by an ultrasonic method (Ekomilk Total^®^ milk analyzer, Cap-Lab, São Paulo, Brazil). Concentrations of total solids were calculated by adding the fat concentration to the defatted dry extract.

The reducing power of milk samples indicates the ability of a sample to scavenge free radicals and inhibit lipid peroxidation, and it was determined according to Zhu et al. [8], with the modifications of Santos et al. [21], and the absorbance was determined in a UV-VIS spectrophotometer. Reducing power was expressed as gallic acid equivalents (GAE, mg/L). Total antioxidant capacity (TAC) of the milk samples was determined as described by Rufino et al. [22], with the addition of radical ABTS+-(2,2-azinobis-(3-ethyl-benzothiazolin-6-sulfonic acid)) to the extract. TAC was expressed as Trolox^®^ (Sigma-Aldrich Co., St. Louis, MO, USA) equivalents (µM TE/L).

Analysis of conjugated diene hydroperoxides (CD) in milk was performed according to the methodology described by Kiokias et al. [23], and the absorbance was determined at 232 nm in a UV-VIS spectrophotometer, expressed as mmol/kg fat. Analysis of thiobarbituric acid-reactive substance (TBARS) content was performed according to Vyncke [24]. Values were expressed as mg of malonaldehyde/L.

Total lipids were extracted from feed samples with chloroform, methanol, and water in a 2:2:1.8 ratio, as described by Bligh and Dyer [25]. Total lipids were extracted from milk samples with chloroform, methanol, and water in a 2:1:1 ratio, as described by Folch et al. [26]. The transesterification was performed through a basic methylation of samples, according to the methodology of Hartman and Lago [27], modified by Maia and Rodriguez-Amaya [28].

Fatty acid methyl esters were separated by a gas chromatograph equipped with a fused silica capillary column (CP-7420, Select FAME, 100 m × 0.25 mm diameter and 0.25 μm cyanopropyl) and flame ionization detector [29]. The gas flow was 1.2 mL/min for the entrainment gas (H2), 30 mL/min for the auxiliary gas (N2), 35 mL/min for H2, and 350 mL/min for the synthetic gas. The volume of injection was 2.0 μL, using a 1:80 sample split. The temperatures of the injector and the detector were 240 °C. The column temperature was 165 °C for 7 min, followed by the first heating ramp from 4 °C/min to 185 °C, which was held for 12 min, and then followed by another heating ramp from 6 °C/min until 235 °C was reached, totaling 25 min of analysis. The peak areas and retention times were determined using the ChromQuest software 5.0. The fatty acid methyl esters were identified by comparing the retention times with the standard methyl esters 18,919 (Sigma-Aldrich Co., St. Louis, USA). The fatty acids were quantified against tricosanoic acid methyl ester (Sigma-Aldrich Co., St. Louis, USA) as an internal standard, as described by Joseph and Ackman [30]. Theoretical correction factors were used to determine the fatty acid (FA) concentrations, as described by Visentainer [31]. The concentrations of FA were calculated in mg/g of total fatty acids (mg FA/g total FA).

### 2.4. Statistical Analyses

The activity of Δ9-desaturase enzyme was estimated according to the following equations, proposed by Schennink et al. [32]:

*cis*-9 14:1/14:0 = [*cis*-9 C14:1/(C14:0 + *cis*-9 C14:1)] × 100 (1)

*cis*-9 16:1/16:0 = [*cis*-9 C16:1/(C16:0 + *cis*-9 C16:1)] × 100 (2)

*cis*-9 18:1/18:0 = [*cis*-9 C18:1/(C18:0 + *cis*-9 C18:1)] × 100 (3)

Data were analyzed with the GLM procedure of SAS (Statistical Analysis System, 9.0, Cary, NC, USA) according to a 4 × 4 Latin square design, with a 2 × 2 factorial arrangement of the treatments. Flaxseed oil supplementation in the diet (Oil), vitamin E supplementation (Vit. E), and the interaction between both supplementations (Interaction). The model assumptions were met, and data followed a normal distribution, as assessed by the Shapiro–Wilk test. No experimental unit or observations were removed from our model after checking for outlier and influential points. The statistical model was as follows:

Y_ijkl_ = μ + A_i_ + P_j_ + O_k_ + V_l_ + O_k_ × V_l_ + ɛ_ijkl_ (4)

where Y_ijkl_ = dependent variable, μ = general mean, A_i_ = effect of animal _i_ ranging from 1 to 4, P_j_ = effect of period _j_ ranging from 1 to 4, O_k_ = effect of presence or absence flaxseed oil _k_, V_l_ = effect of presence or absence of vitamin E _l_, O_k_ × V_l_ = effect of the interaction between flaxseed oil and vitamin E, and ɛ_ijk_ = random error. The statistical model to determine IVDMD was as follows:

Y_ijkl_ = μ + D_i_ + O_j_ + V_k_ + O_j_ × V_k_ + ɛ_ijk_ (5)

where Y_ijk_ = dependent variable, μ = general mean, D_i_ = effect of day _i_ ranging from 1 to 3, O_j_ = effect of presence or absence flaxseed oil _j_, V_k_ = effect of presence or absence of vitamin E _k_, O_j_ × V_k_ = effect of the interaction between flaxseed oil and vitamin E, and ɛ_ijk_ = random error. Differences were declared significant at *p* ≤ 0.05 and tendencies if *p* > 0.05 and *p* ≤ 0.10.

## 3. Results

There was no interaction (*p* > 0.05) between flaxseed oil and vitamin E supplementation to the diet on DM and nutrient intake or digestibility or on milk yield (actual, 4% fat-corrected, energy-corrected), milk nutrients yield, or fatty acid composition. Flaxseed oil supplementation in the diet increased EE intake (*p* = 0.01) (Table 2). The intake of the other nutrients was similar among treatments (*p* > 0.05). Flaxseed oil supplementation at 25 g/kg DM reduced the total digestibility of DM (*p* = 0.02), organic matter (OM) (*p* < 0.01), NDF (*p* < 0.01), ADF (*p* < 0.01), and IVDMD (*p* < 0.01). In addition, flaxseed oil supplementation increased the EE digestibility (*p* < 0.01) (Table 2) and tended to increase the digestibility of CP (*p* = 0.07). On the other hand, the vitamin E supplementation increased (*p* = 0.02) the digestibility of NDF and tended to increase the digestibility of DM (*p* = 0.08) and OM (*p* = 0.07).

Flaxseed oil supplementation reduced milk fat concentration (*p* = 0.04) (Table 3), which consequently increased milk density (*p* = 0.02) and tended to reduce total solid concentration (*p* = 0.08). Vitamin E supplementation did not affect (*p* > 0.05) milk composition or density. Defatted dry extract concentration, milk yield, and milk nutrients yield did not differ (*p* > 0.05) among treatments.

There were tendencies for interactions between flaxseed oil and vitamin E to increase the reducing power (*p* = 0.07) and TAC (*p* = 0.07) of the milk. When adding vitamin E to diets, a significant increase (*p* = 0.01) was observed in milk reducing power (Table 3), while the flaxseed oil supplementation increased the content of CD and TBARS in milk (*p* < 0.01). However, vitamin E improved oxidative stability, because it reduced the concentration of TBARS in milk (*p* = 0.02).

The treatment with additional flaxseed oil reduced (*p* < 0.05) the concentrations of short-chain FA (C8:0, C10:0, and C12:0) and medium-chain FA (C14:0, C14:1n-9, C15:0, C16:0, C16:1n-7, and C17:0), and it increased the concentrations of the long-chain fatty acids (C18:2n-6) in milk fat (Table 4). Thus, flaxseed oil supplementation reduced the total concentration of short-chain FA (*p* = 0.04) (Table 5) and medium-chain FA in milk fat (*p* < 0.01). Flaxseed oil supplementation had a tendency to increase the concentration of long-chain FA (*p* = 0.06), and it increased C18:0 (*p* = 0.02), *trans*-11 C18:1 (*p* = 0.04), *trans*-6 18:2 (*p* < 0.01), C18:3n-3 (*p* = 0.01), and C22:6n-3 (*p* = 0.05), reduced the n-6/n-3 ratio (*p* = 0.01) from 9.3 to 2.4, and increased the quantity of PUFAs (*p* = 0.03) and n-3 (*p* = 0.01).

The vitamin E supplementation increased FA C14:1n-9 (*p* = 0.04), C15:0 (*p* = 0.04), C16:1n-9 (*p* = 0.03), C16:1n-7 (*p* = 0.02), C17:0 (*p* = 0.02), C18:2n-6 (*p* = 0.02), and C21:0 (*p* = 0.02) in milk fat, as well as increasing the concentrations of FA n-6 (*p* < 0.01) and PUFA (*p* = 0.04) (Table 5). Total CLA concentration was not affected (*p* > 0.05) by the treatments; however, lipid supplementation tended to increase the isomer *cis*-c-11 C18:2 (*p* = 0.06).

Flaxseed oil supplementation reduced the activity of the Δ9-desaturase enzyme in milk on the FA C14:0 (*p* < 0.01), C16:0 (*p* = 0.03), and C 18:0 (*p* = 0.01). However, vitamin E increased the Δ9-desaturase activity on the FA C14:0 (*p* < 0.01) and C 16:0 (*p* < 0.01).

## 4. Discussion

The addition of flaxseed oil into the diet increased EE concentration as well as EE intake and digestibility. Although the EE content and flaxseed oil supplementation levels of the diets in the present study did not exceed the levels used in the study of Benchaar et al. [34] with dairy cows, in which no adverse effects on digestibility were observed, the total digestibility of NDF, ADF, and ruminal in vitro DM digestibility of oil supplemented diets decreased. This reduction in the digestibility of the fibrous fraction led to a decrease in total DM and OM digestibility (Table 2). It has been shown that PUFA are deleterious to microorganisms [11] and mainly affect those that degrade the cell wall, since these FA have a cytotoxic effect [35]. The reduction in the total digestibility of NDF and ADF differs from the findings of Yoshimura et al. [36], who added the same amount of flaxseed oil into the diets of dairy cows and did not have differences in these parameters.

Naziroğlu et al. [37], when evaluating the addition of vitamin E in ruminal fermentation in vitro, observed an increase in acetate concentration, as well as in protozoa population, that plays an important role in cellulose degradation. However, the vitamin E mechanism in ruminal microorganisms is not known yet [38].

The reduction in milk fat concentration of buffaloes supplemented with flaxseed oil suggests a reduction in de novo synthesis, probably associated with the observed reduction in NDF digestibility. According Bauman and Griinari [39], when acetate and beta hydroxybutyrate concentrations decrease, which are main precursors for de novo synthesis in the mammary gland, there is a propensity towards milk fat depression (MFD). Acetate and beta hydroxybutyrate come from ruminal fiber fermentation; therefore, reduced fiber digestibility is strongly related to MFD. Urrutia and Harvatine [40] demonstrated that increasing the acetate supply to lactating cows increased milk fat synthesis. Another possibility for MFD is the increase in fatty acids from partial ruminal biohydrogenation, which is known to negatively impact the expression of genes involved in de novo synthesis, such as FA *trans*-10, *cis*-12 18:2 [12]. The FA *trans*-10, *cis*-12 C18:2 is derived from the ruminal isomerization of the C18:2n-6, FA present in flaxseed oil (137 mg/g of total FA). However, flaxseed oil supplementation did not affect this FA concentration in milk. Lower concentrations of short and medium-chain FA (*p* = 0.04 and *p* < 0.01, respectively) in milk fat (Table 5) confirm the MFD in diets with flaxseed oil supplementation. Santillo et al. [4] observed that short and medium-chain FA concentrations decreased and there was no difference in milk fat concentration of buffalo supplemented with flaxseed in diets with different crude protein levels.

The vitamin E supplementation in the diet increased the reducing power in milk, confirming the notion that dietary antioxidants were partially transferred to the milk, improving its antioxidant capacity. A tendency towards interactions between flaxseed oil and vitamin E for reducing power and TAC (Table 5) probably occurred due to vitamin E being fat-soluble and having better intestinal absorption when added to oil-rich diets. The increase in antioxidants parameters with vitamin E supplementation is interesting because the milk became consequentially enriched.

The increased CD and TBARS content (Table 3) in the milk of buffaloes fed diets with lipid supplementation were probably caused by the increase in PUFA concentration, which are more likely to undergo lipoperoxidation compared to SFA [7]. This effect is due to double bonds, which cause the loss of electrons. In contrast, the reduction in TBARS in milk by vitamin E supplementation confirms the antioxidant capacity (reducing power) of milk and the reduced lipoperoxidation. The reduction in TBARS is important for increasing milk’s chemical stability. The results are similar to Focant et al. [41], who observed that the resistance of milk fat to oxidation improved when dairy cows were supplemented with vitamin E in diets with oil supplementation.

Increased concentrations of the FA C18:3n-3 in milk was probably due to a greater amount of this FA that arrived in abomasum from flaxseed oil supplementation. Flaxseed oil has 483 mg/g of total FA (Table 1), and, when supplemented in the diet, the level was greater than the biohydrogenation capacity of ruminal bacteria. The increased concentrations of the FA *trans*-11 C18:1 and C18:0 in milk fat (Table 4) were due, respectively, to exposure to incomplete or complete ruminal biohydrogenation of C18:3n-3 and C18:2n-6. Santillo et al. [4] observed the same pattern for C18:3n-3, *trans*-11 C18:1, and C18:0 FA in the milk fat of buffaloes supplemented with flaxseed oil in diets with different protein levels. However, the abomasal infusion of flaxseed oil led to a greater increase in C18:3n-3 [42].

Although the apparent digestibility of EE was greater in diets with flaxseed oil supplementation, which could potentially yield more substrate to incorporation in milk fat, this was not observed. This can be explained through the partitioning of polyunsaturated fatty acids that usually are less available to the mammary gland because polyunsaturated fatty acids are primarily transported through phospholipids or cholesterol ester. This mechanism makes these FA less available to the mammary gland, which is a mechanism to protect some essential fatty acids from incorporation in the milk so they can be used by the animal instead [43].

Some studies have shown that the n-6/n-3 ratio is as important as the amount of n-3 FA, because when this ratio increases to 15–20:1, inflammation increases. This is because FA n-6 competes with FA n-3 for the enzymes responsible for desaturation and elongation [5], which can promote a reduction in the synthesis of docosahexaenoic acid and eicosapentanoic acids while increasing the concentration of arachidonic acid, a substrate for the production of pro-inflammatory, vasoconstricting, and pro-aggregating substances [44]. In our study, for diets with flaxseed oil supplementation, the n6/n3 ratio in milk was reduced by approximately 74%. The secretion of *cis*-9, *trans*-11 C18:2 FA in milk occurred by its formation in the rumen, or the action of the enzyme in the mammary gland, which forms this CLA isomer from the *trans*-11 C18:1 previously formed in the rumen [45]. Therefore, due to its health benefits, such as the tendency to increase the concentration of *cis*-9, *trans*-11 C18:2 in milk fat, lipid supplementation was considered beneficial.

Diets rich in PUFA adversely affect the expression of genes responsible for Δ9-desaturase function [46]. This fact explains why, in diets containing flaxseed oil, the activity of Δ9-desaturase enzyme in milk was reduced. Vitamin E increased the activity of Δ9-desaturase enzyme to FA C14:0 and C160, probably due to its role as a cofactor of enzymes responsible for the desaturation of fatty acids [47]. The greater activity of Δ9-desaturase enzyme may promote an increase in unsaturated FA concentration and reduces SFA in milk, further improving milk’s FA profile.

Similar to the present study, Santos et al. [10], supplementing Holstein cows with similar concentrations of flaxseed oil and vitamin E, also observed an increase in the concentrations of PUFA, *cis*-9, *trans*-11 C18:2 FA, and n-3 FA, and reductions in SFA and the n-6/n-3 ratio, as well as a reduction in milk fat concentration.

## 5. Conclusions

We can conclude that there was no interaction between flaxseed oil and vitamin E supplementation in water buffaloes; however, flaxseed oil supplementation increased PUFA in the milk. On the other hand, vitamin E supplementation increased the antioxidant capacity of milk and decreased the oxidation products. Thus, in general, both supplementations were advantageous in making the milk more desirable to humans from a health perspective.

## Figures and Tables

**Table 1:** Ingredient and chemical composition of the experimental diets.

Item	No Flaxseed Oil		With Flaxseed Oil	
	No Vitamin E	With Vitamin E	No Vitamin E	With Vitamin E
Ingredients (g/kg DM, unless otherwise stated)				
Corn silage	700	700	700	700
Ground corn	112.9	112.9	40.0	40.0
Soybean meal (48%, solvent-extracted)	61.3	61.3	53.3	53.3
Wheat meal	92.6	92.6	148.5	148.5
Flaxseed oil ^1^	-	-	25.0	25.0
Vitamin E (IU/kg DM) ^2^	-	375	-	375
Mineral and vitamin supplement ^3^	20.0	20.0	20.0	20.0
Limestone	8.8	8.8	8.8	8.8
Urea	4.0	4.0	4.0	4.0
Ammonium sulfate	0.4	0.4	0.4	0.4
Chemical composition				
Dry matter (g/kg fresh weight)	472.6	472.6	476.4	476.4
Organic matter (g/kg DM)	928.2	928.2	922.5	922.5
Crude protein (g/kg DM)	116.3	116.3	116.7	116.7
Ether extract (g/kg DM)	26.4	26.4	50.7	50.7
Neutral detergent fiber (g/kg DM)	497.9	497.9	507	507
Acid detergent fiber (g/kg DM)	252.9	252.9	256.7	256.7
Non-fibrous carbohydrates (g/kg DM)	299.1	299.1	263.2	263.2
Net energy for lactation (Mcal/kg DM) ^4^	1.46	1.46	1.52	1.52
Fatty acids (g/kg DM of diet)				
16:0	5.79	5.79	7.28	7.28
18:0	0.45	0.45	1.77	1.77
18:1n-9	5.56	5.56	10.51	10.51
18:2n-6	3.86	3.86	7.01	7.01
18:3n-3	0.49	0.49	13.56	13.56
n-6	4.45	4.45	7.58	7.58
n-3	0.49	0.49	12.56	12.56
n-6/n-3	9.04	9.04	0.60	0.60
Flaxseed oil fatty acid composition (mg/g of total fatty acids)				
16:0	62			
18:0	53			
18:1n-9	201			
18:2n-3	137			
18:3n-3	483			

DM = dry matter; ^1^ = Lino Oil, Cisbra, Panambi, RS, Brazil; ^2^ = Microvit E Promix 500,000 IU/kg, Adisseo; ^3^ = mineral and vitamin supplement (per kilogram of product): calcium 145 g, phosphorous 51 g, sulfur 20 g, magnesium 33 g, potassium 28 g, sodium 93 g, cobalt 30 mg, copper 400 mg, chrome 10 mg, iron 2000 mg, iodine 40 mg, manganese 1350 mg, selenium 15 mg, zinc 1700 mg, fluorine 510 mg, vitamin A 135,000 IU, vitamin D3 68,000 IU, vitamin E 450 IU; ^4^ = calculated according to the NRC (2001; p. 13) [15].

**Table 2:** IVDMD, dry matter (DM) and nutrient intake (kg/day), and apparent total tract digestibility (kg/kg) of lactating buffaloes fed diets containing flaxseed oil and/or vitamin E (IVDMD).

Item	No Flaxseed Oil		With Flaxseed Oil		SEM	*p* Value ^1^		
	No Vit. E	With Vit. E	No Vit. E	With Vit. E		Oil	Vit. E	Interaction
**Intake (kg DM/day)**								
Dry matter	13.30	13.59	12.76	12.88	0.306	0.35	0.75	0.89
Organic matter	14.20	11.33	10.98	12.35	0.305	0.15	0.72	0.87
Crude protein	1.59	1.61	1.59	1.58	0.052	0.79	0.90	0.80
NDF	6.42	6.61	6.26	6.36	0.173	0.55	0.68	0.89
ADF	3.34	3.33	3.24	3.17	0.091	0.42	0.79	0.88
Ether extract	0.37	0.38	0.63	0.66	0.039	0.01	0.62	0.98
**Total Apparent Digestibility (g/kg of DM)**								
Dry matter	684.3	690.2	666.8	679.0	3.41	0.02	0.08	0.49
Organic matter	697.3	702.1	668.3	680.9	4.43	<0.01	0.07	0.37
Crude protein	667.7	650.9	692.4	698.0	8.84	0.07	0.75	0.53
NDF	582.7	605.9	551.9	570.4	7.94	<0.01	0.02	0.73
ADF	586.3	573.7	530.5	535.0	11.40	<0.01	0.75	0.51
Ether extract	853.0	847.7	897.3	925.1	11.81	<0.01	0.32	0.16
IVDMD (kg/kg)	566.0	581.9	501.2	507.3	12.03	<0.01	0.59	0.81

^1^ Probability of significant factorial effects. Effects tested were as follows: Oil = flaxseed oil supplementation; Vit. E = vitamin E supplementation; Interaction = interaction between flaxseed oil and vitamin E. NDF = neutral detergent fiber; ADF = acid detergent fiber; IVDMD = in vitro dry matter digestibility [16].

**Table 3:** Composition and antioxidant capacity of milk from lactating buffaloes fed diets containing flaxseed oil and/or vitamin E.

	No Flaxseed Oil		With Flaxseed Oil		SEM	*p* Value ^1^		
	No Vit. E	With Vit. E	No Vit. E	With Vit. E		Oil	Vit. E	Interaction
Density (g/mL)	1.0318	1.0313	1.0325	1.0321	<0.001	0.02	0.82	0.45
Milk yield (kg/day)								
Actual	6.53	5.88	6.91	6.96	0.540	0.18	0.55	0.50
4% Fat-corrected ^2^	8.86	8.40	8.77	9.18	0.678	0.66	0.97	0.58
Energy-corrected ^3^	9.39	8.83	9.44	9.82	0.727	0.51	0.91	0.55
Yield (kg/day)								
Fat	0.42	0.40	0.40	0.43	0.031	0.93	0.88	0.62
Protein	0.26	0.23	0.28	0.28	0.022	0.12	0.60	0.45
Lactose	0.32	0.29	0.34	0.35	0.026	0.12	0.64	0.43
Defatted dry extract	0.62	0.57	0.67	0.68	0.052	0.11	0.60	0.45
Total solids	1.04	0.97	1.070	1.11	0.082	0.32	0.82	0.51
Concentration (g/kg)								
Fat	64.84	68.20	59.24	61.23	1.672	0.04	0.29	0.77
Protein	39.89	39.83	40.07	40.42	0.114	0.18	0.59	0.45
Lactose	48.96	48.83	49.21	49.92	0.181	0.13	0.48	0.31
Defatted dry extract	96.13	95.88	96.88	97.78	0.323	0.11	0.67	0.45
Total solids	161.0	164.1	156.1	159.0	1.645	0.08	0.25	0.96
Antioxidants								
Reducing power ^4^	20.67	22.50	19.29	27.71	1.239	0.25	0.01	0.07
TAC (TE µM/L)	236.6	222.2	230.1	249.9	5.780	0.23	0.75	0.07
Oxidation products								
TBARS (MDA mg/L)	27.69	24.73	39.15	28.59	0.337	< 0.01	0.02	0.13
CD (mmol/kg fat)	20.34	24.37	42.20	38.85	2.665	< 0.01	0.87	0.12

^1^ Probability of significant factorial effects. Effects tested were as follows: Oil = flaxseed oil supplementation; Vit. E = vitamin E supplementation; Interaction = interaction between flaxseed oil and vitamin E. ^2^ 4% fat-corrected (kg/d) = (0.4 × milk yield (kg/d)) + (15.0 × fat yield (kg/d)) [15]; ^3^ ECM (kg/d) = (0.327 × kg of milk) + (12.95 × kg of fat) + (7.2 × kg of protein) [33]; ^4^ expressed as gallic acid equivalents (mg/L). ECM = energy-corrected milk; TAC = total antioxidant capacity; TE = Trolox equivalents; TBARS = thiobarbituric acid reactive substances; MDA = malonaldehyde; CD = conjugated diene hydroperoxides.

**Table 4:** Milk fatty acid composition (mg/g total FA) of lactating buffaloes fed diets containing flaxseed oil and/or vitamin E.

Fatty Acid	No Flaxseed Oil		With Flaxseed Oil		SEM	*p* Value ^1^		
	No Vit. E	With Vit. E	No Vit. E	With Vit. E		Oil	Vit. E	Interaction
8:0	8.73	5.96	4.49	3.31	0.63	<0.01	0.03	0.28
10:0	17.95	14.67	9.38	13.75	1.32	0.17	0.87	0.26
11:0	0.64	0.83	0.50	0.51	0.06	0.08	0.37	0.41
12:0	22.33	21.72	13.60	17.03	1.27	0.03	0.56	0.41
13:0	0.85	0.64	0.62	0.69	0.07	0.52	0.65	0.35
14:0	98.88	96.50	75.04	70.80	4.41	0.02	0.67	0.90
14:1n-9	7.98	9.56	4.22	6.52	0.69	<0.01	0.04	0.64
14:1n-7	4.20	4.92	3.49	4.26	0.26	0.11	0.09	0.95
15:0	8.02	9.22	5.00	6.43	0.52	<0.01	0.04	0.82
15:1n-7	3.49	3.30	2.85	3.20	0.21	0.25	0.80	0.39
16:0	251.66	259.97	194.45	198.03	9.07	<0.01	0.66	0.86
16:1n-11	0.48	0.50	0.58	0.76	0.05	0.09	0.29	0.40
16:1n-9	1.52	1.73	1.40	1.76	0.06	0.67	0.03	0.53
16:1n-7	14.53	20.39	8.83	10.65	1.34	<0.01	0.02	0.17
16:1n-5	2.41	3.07	2.26	2.60	0.17	0.34	0.15	0.61
17:0	4.42	5.44	3.62	4.54	0.23	0.03	0.02	0.87
17:1n-9	1.41	1.78	1.98	1.21	0.19	0.99	0.56	0.14
18:0	87.82	88.54	118.47	125.02	5.88	0.02	0.74	0.79
*trans*-11 18:1	5.14	5.87	11.09	11.14	1.25	0.04	0.87	0.88
*cis*-9 18:1	161.96	195.27	180.72	185.37	7.31	0.75	0.21	0.33
18:1n-7	3.09	4.53	3.61	2.66	0.41	0.42	0.77	0.18
*trans*-6 18:2	1.52	1.57	3.53	4.75	0.50	<0.01	0.16	0.19
18:2n-6	15.91	20.10	13.41	14.84	8.49	<0.01	0.02	0.17
18:3n-3	3.83	2.81	7.68	12.24	1.45	0.01	0.40	0.20
*cis*-9, *trans*-11 18:2	3.33	3.40	4.12	5.12	0.34	0.06	0.37	0.43
*trans*-10, *cis*-12 18:2	2.00	1.52	1.40	1.77	0.11	0.42	0.78	0.08
20:1n-9	1.32	0.88	1.93	0.97	0.13	0.38	0.24	0.17
20:3n-6	0.79	0.67	0.40	0.41	0.08	0.10	0.75	0.70
20:4n-6	0.93	1.57	1.58	0.79	0.19	0.86	0.85	0.11
20:5n-3	0.24	0.47	0.21	0.39	0.07	0.66	0.14	0.83
21:0	0.13	0.44	0.11	0.29	0.05	0.36	0.02	0.44
22:0	0.75	0.68	0.74	0.82	0.10	0.79	0.97	0.76
22:4n-6	0.71	1.16	0.85	1.42	0.18	0.44	0.08	0.81
22:5n-6	0.23	0.38	0.46	0.22	0.06	0.77	0.70	0.12
22:6n-3	0.16	0.39	0.69	1.14	0.16	0.05	0.40	0.69
24:0	0.46	0.49	0.86	0.40	0.11	0.43	0.29	0.23
24:1n-9	0.60	0.84	1.12	0.69	0.09	0.33	0.60	0.09

^1^ Probability of significant factorial effects. Effects tested were as follows: Oil = flaxseed oil supplementation; Vit. E = vitamin E supplementation; Interaction = interaction between flaxseed oil and vitamin E.

**Table 5:** Concentration of total CLA, short, medium, and long-chain fatty acids, n-6, n-3 (mg/g total lipids), n-6/n-3 ratio, index of atherogenicity (IA), and thrombogenicity (IT) in milk from lactating buffaloes fed diet containing flaxseed oil and/or vitamin E.

Fatty Acid	No Flaxseed Oil		With Flaxseed Oil		SEM	*p* Value ^1^		
	No Vit. E	With Vit. E	No Vit. E	With Vit. E		Oil	Vit. E	Interaction
Total CLA ^2^	5.33	4.91	5.52	6.90	0.51	0.16	0.33	0.91
Short-chain FA ^3^	50.48	43.82	28.59	35.29	3.03	0.04	0.99	0.30
MCFA ^4^	399.0	416.4	303.7	310.7	15.17	< 0.01	0.57	0.81
Long-chain FA ^5^	290.9	338.6	351.8	370.4	12.07	0.06	0.23	0.64
SFA ^6^	502.6	505.1	426.8	441.6	14.77	0.08	0.80	0.86
MUFA ^7^	208.1	252.6	223.1	231.8	8.95	0.85	0.13	0.29
PUFA ^8^	29.62	34.00	34.31	43.08	1.70	0.03	0.04	0.43
n-6	20.07	25.44	20.22	22.43	1.14	0.20	<0.01	0.16
n-3	4.22	3.66	8.57	13.76	1.62	0.01	0.30	0.21
n-6/n-3	9.65	8.94	2.64	2.24	1,51	0.01	0.74	0.93
Δ9-desaturase enzyme activity								
*cis*-9 14:1/14:0	8.13	6.89	6.41	8.60	0.46	<0.01	0.01	0.06
*cis*-9 16:1/16:0	7.98	6.91	6.69	8.20	0.40	0.03	<0.01	0.24
*cis*-9 18:1/18:0	66.61	60.24	62.76	64.09	1.27	0.01	0.47	0.29

^1^ Probability of significant factorial effects. Effects tested were as follows: Oil = flaxseed oil supplementation; Vit. E = vitamin E supplementation; Interaction = interaction between flaxseed oil and vitamin E. CLA = conjugated linoleic acid; FA = fatty acids; MCFA = medium-chain fatty acids; SFA = saturated fatty acid; MUFA = monounsaturated fatty acid; PUFA = polyunsaturated fatty acid. ^2^ Total CLA = (*cis*-9, *trans*-11 18:2 + *trans*-10, *cis*-12 18:2). ^3^ Short-chain FA = ∑(FA ≤ 13C). ^4^ Medium-chain FA = ∑(FA ≥ 14C and ≤17C). ^5^ Long-chain FA = ∑(FA ≥ 18C). ^6^ SFA = ∑(saturated fatty acid). ^7^ MUFA = ∑(monounsaturated fatty acids). ^8^ PUFA = ∑(polyunsaturated fatty acid).
